# A Novel Approach to Assess Balneotherapy Effects on Musculoskeletal Diseases—An Open Interventional Trial Combining Physiological Indicators, Biomarkers, and Patients’ Health Perception

**DOI:** 10.3390/geriatrics8030055

**Published:** 2023-05-16

**Authors:** Jani Silva, José Martins, Cristina Nicomédio, Catarina Gonçalves, Cátia Palito, Ramiro Gonçalves, Paula Odete Fernandes, Alcina Nunes, Maria José Alves

**Affiliations:** 1 AquaValor—Centro de Valorização e Transferência de Tecnologia da Água, Rua Dr. Júlio Martins, nº1, 5400-342 Chaves, Portugal; jani.silva@aquavalor.pt (J.S.); jose.martins@aquavalor.pt (J.M.); catia.palito@aquavalor.pt (C.P.); ramiro@utad.pt (R.G.); 2 Molecular Oncology and Viral Pathology Group, Research Center of IPO Porto (CI-IPOP)/Pathology and Laboratory Medicine Department, Clinical Pathology SV/RISE@CI-IPOP (Health Research Network), Portuguese Oncology Institute of Porto (IPO Porto)/Porto Comprehensive Cancer Centre (Porto.CCC), 4200-072 Porto, Portugal; 3 INESC TEC-Institute for Systems and Computer Engineering, Technology and Science, Campus da Faculdade de Engenharia da Universidade do Porto, Rua Dr. Roberto Frias, 4200-465 Porto, Portugal; 4 Instituto Politécnico de Bragança, Campus de Santa Apolónia, 5300-253 Bragança, Portugal; 5 Unidade Local de Saúde do Nordeste EPE, Unidade Hospitalar de Mirandela, 5370-210 Mirandela, Portugal; nicoredio@hotmail.com; 6 Department of Engineering, School of Sciences and Technologies, Universidade de Trás-os-Montes e Alto Douro, 5000-801 Vila Real, Portugal; 7 UNIAG, Instituto Politécnico de Bragança, 5300-271 Bragança, Portugal; pof@ipb.pt (P.O.F.); alcina@ipb.pt (A.N.); 8 Centro de Investigação de Montanha (CIMO), Instituto Politécnico de Bragança, 5300-271 Bragança, Portugal

**Keywords:** musculoskeletal diseases, natural mineral water, balneotherapy, IL-6, health-related quality of life, quality of sleep, wearable sensing device

## Abstract

The present study aimed to evaluate whether a 14-day period of balneotherapy influences the inflammatory status, health-related quality of life (QoL) and quality of sleep, underlying overall health state, and clinically relevant benefits of patients with musculoskeletal diseases (MD). The health-related QoL was evaluated using the following instruments: 5Q-5D-5L, EQ-VAS, EUROHIS-QOL, B-IPQ, and HAQ-DI. The quality of sleep was evaluated by a BaSIQS instrument. Circulating levels of IL-6 and C-reactive protein (CRP) were measured by ELISA and chemiluminescent microparticle immunoassay, respectively. The smartband, Xiaomi MI Band 4, was used for real-time sensing of physical activity and sleep quality. MD patients improved the health-related QoL measured by 5Q-5D-5L (*p* < 0.001), EQ-VAS (*p* < 0.001), EUROHIS-QOL (*p* = 0.017), B-IPQ (*p* < 0.001), and HAQ-DI (*p* = 0.019) after balneotherapy; the sleep quality was also improved (BaSIQS, *p* = 0.019). Serum concentrations of IL-6 were markedly decreased after the 14-day balneotherapy (*p* < 0.001). No statistically significant differences were observed regarding the physical activity and sleep quality data recorded by the smartband. Balneotherapy may be an effective alternative treatment in managing the health status of MD patients, with a decrease in inflammatory states, along with positive effects on pain reduction, patient’s functionality, QoL, quality of sleep, and disability perception status.

## 1. Introduction

Musculoskeletal diseases (MD) have been described as disorders/conditions in which a portion of the musculoskeletal system is injured or has deteriorated over time. Symptoms include acute or chronic, focal or diffuse pain, dysfunction, or discomfort in the bones, joints, muscles, or surrounding structures. Osteoarthritis, fibromyalgia, rheumatoid arthritis, and low-back pain are the most prevalent musculoskeletal illnesses [1,2,3]. These disorders have been characterized by the development of painful symptoms at the joint level and anatomical-functional impairments with systemic involvement, resulting in reduced quality of life (QoL) and an increased risk of morbidity [4]. According to recent statistics from the Global Burden of Disease (2019), about 1.71 billion individuals worldwide suffer from musculoskeletal conditions. Although the prevalence of the abovementioned disorders varies by age and diagnosis, they tend to affect people of all ages worldwide [1].

Natural mineral water-based therapies, particularly balneotherapy [5], evolved from an initial state based only on empirical knowledge (very much supported by intangible heritage) to modern approaches supported by the progressive systematization of scientific knowledge combined with the experience drawn from their widespread use [6,7,8]. The renewed vision of using natural mineral water has brought conceptual clarification towards the efficiency and effectiveness of the therapeutic techniques and the inherent health and wellness effects [9,10,11,12,13]. Despite the fact that balneotherapy has been described as a non-pharmacological add-on for the adjuvant treatment of numerous MDs, the underlying biological mechanisms by which it mitigates MD-related symptoms are not entirely elucidated.

Indeed, in recent years, several relevant studies, including randomized clinical trials, meta-analyses, and systematic reviews, have improved this vision of balneotherapy as an alternative treatment approach to different MDs [14,15,16,17,18]. The effects of balneotherapy are probably related to the temperature, physicochemical, and microbial signature of natural mineral waters. This type of therapy triggers a set of biological, physiological, and perceptional responses involved in the pathophysiology of MD, namely, (i) neuroendocrine reaction that increases serum levels of opioid peptides, such as endorphins, (ii) change in the circulating levels of prostaglandins, leukotrienes, metalloproteinases, (iii) change in the circulating levels of inflammatory biomarkers, (iv) improve physical function, and (v) QoL indicators [10,19,20,21,22,23,24]. Additionally, the thermal spa environment provides a variety of rehabilitative and therapeutic interventions, including physical therapy, therapeutic exercise (e.g., hydrokinetic therapies, functional mobility, or functional training), and health-related preventive and educational actions for a healthy lifestyle approach.

Hence, the main objective of this study was to establish a novel multi-perspective approach focused on evaluating the potential impacts that a 14-day balneotherapy treatment plan might have on the inflammatory status, physiological indicators, individual perception of functionality, emotional and social elements, and QoL, thus underlying the overall health and wellness state, and clinically relevant benefits in patients with MD.

## 2. Materials and Methods

### 2.1. Study Design, Patients, and Sample Collection

**Figure 1:**
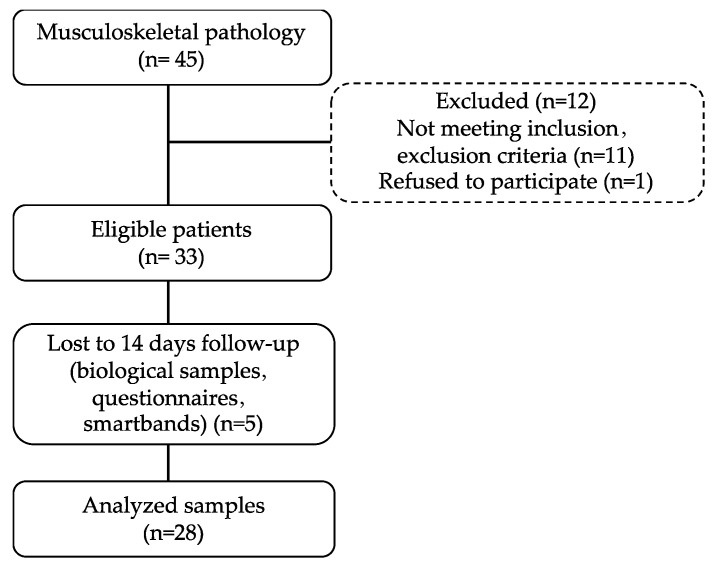
Flow chart of the recruitment and 14 days follow-up process.

We conducted an open interventional trial to evaluate the effects of 14 days of balneotherapy in a group of 28 volunteer MD patients (mean age ± standard deviation (SD), 56.071 ± 9.641) recruited from Termas de Chaves spa, in collaboration with Chaves’ Primary Health Care Unit, from August 2021 to September 2021. All volunteers who fulfilled the inclusion/exclusion criteria were accepted after being formally informed about the study. Thirty-three patients who met the eligibility criteria were enrolled in the study, and five patients withdrew due to a loss to follow-up (Figure 1).

The inclusion criteria were as follows: (a) diagnosis of osteophytosis and/or herniated discs and (b) medical prescription for treatments. The exclusion criteria were as follows: (a) having comorbidities (the study participants were selected alongside *the Termas de Chaves* spa medical staff, who performed an initial diagnosis and ensured that the abovementioned participants had no known comorbidities); (b) having undergone balneotherapy or physiotherapy in the last 12 months; (c) being on non-steroidal anti-inflammatory drugs; (d) pregnant women and individuals under 18 years of age; (e) lost to 14 days follow-up.

**Figure 2:**
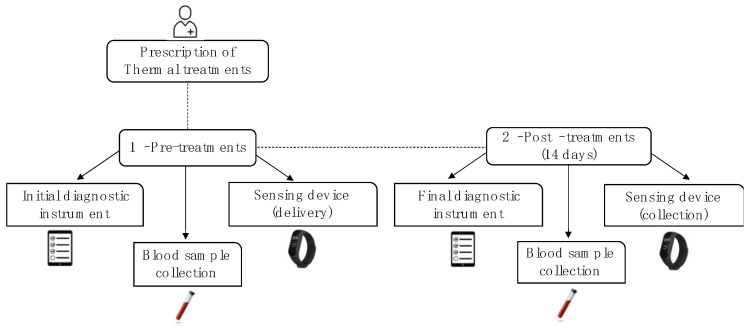
Workflow of procedures according to pre- and post-balneotherapy. 1—pre-treatments (baseline): initial diagnostic instrument (self-administered online questionnaires) and blood sample collection; patients started to use the sensing device (smartband). 2—post-treatments: final diagnostic instrument (self-administered online questionnaires) and blood sample collection; patients handed over the smartband for data extraction.

The study’s objectives were presented to the voluntary participants through an oral presentation that took place in *Termas de Chaves* spa in collaboration with physicians of Chaves’ Primary Health Care Unit. This presentation focused on clarifying the workflow of the study, taking into account the two moments of assessment, pre- and post-balneotherapy (Figure 2).

In order to guarantee anonymity and confidentiality, each participant was identified by a unique alphanumeric code. Study participants completed a comprehensive self-administered online questionnaire with information on sociodemographic data and on scales assessing the QoL, quality of sleep, illness perception, and health assessment. Baseline evaluation and sampling were performed before the first session of balneotherapy. Post-treatment sampling was carried out a day after the last session of balneotherapy.

Peripheral venous blood samples were obtained between 8:00 a.m. and 9:00 a.m. (in a fasted state) with a standard technique (antecubital vein sterile puncture) and collected in serum-separating tubes. Serum samples were separated by centrifugation (2500 rpm for 15 min) and stored in aliquots at −80 °C until analysis.

### 2.2. Balneotherapy

The natural mineral water of Termas de Chaves spa emerges at 77.0 °C. The water has been characterized as gasocarbonic, sodium bicarbonate, fluoride-based, and more mineralized, and contains bicarbonate, chloride, sodium, and potassium as predominant ions (Table 1).

MD patients received a daily morning balneotherapy session for 14 consecutive days, according to the therapeutic procedure implemented in *Termas de Chaves* spa. Daily sessions consisted of a full immersion bath at 36–37 °C, hydrokinesitherapy in a heated swimming pool at 34 °C in which a set of therapeutic exercises were completed, along with Vichy shower/massage (this therapy contains five strategically positioned showers with hot water (T = 39 °C) and associated massage for 15–20 min) in a total 90-min treatment time. Hydrokinesitherapy consists of performing exercises in an aquatic context, initially as segmental motions, afterward coupled or alternated with global and functional exercises (e.g., isometric or isotonic muscle stretching, walking, and swimming) [4,25,26]. All the abovementioned treatments have been performed by a physiotherapist, thus ensuring the proper application of the established therapeutic procedures.

**Table 1:** Principal physicochemical properties and chemical composition of natural mineral water of *Termas de Chaves* spa [27,28].

Physicochemical Properties	
Conductivity (to 20 °C)	2320 µS/cm
pH (to 20 °C)	6.9
Dry residue to 180 °C	1744 mg/L
Hardness (to p.p. 10^5^ CaCO_3_)	7.4 mg/L CaCO_3_
Alkalinity (mL/L de HCl 0.1 M)	288.9 mL/L HCl 0.1 M
**Anions**	mg/L
HCO_3_^−^	1762
Cl^−^	36
SO_4_^2−^	21
F^−^	8.3
PO_4_^3−^	0.22
**Cations**	mg/L
Na^+^	623
K^+^	65
Ca^2+^	22
Mg^2+^	5.5
Fe^2+^	0.17
**Mineral concentration (mg/L)**	2625
CO_2_ dissolved (mg/L)	600

### 2.3. Instruments

Patients’ perceived QoL, functional capacity, illness perception, and sleep quality were assessed by the validated 5Q-5D-5L, EQ-VAS, EUROHIS-QOL-8, BaSIQS, HAQ-DI, and B-IPQ scales.

The 5Q-5D-5L is a generic instrument for measuring health-related quality of life (HRQoL) based on a classification system that describes health in five dimensions: mobility, personal care, usual activities, pain/discomfort, and anxiety/depression, where each of these dimensions has five levels of severity [29,30]. Higher values indicate better QoL. The internal consistency, measured by the Cronbach α coefficient, was 0.836 (0.750–0.900). The second part of the EQ-ED-5L, called EQ-VAS, measures the health status, recorded in a continuous quantitative scale ranging from 0 (worst imaginable health status) to 100 (best imaginable health status) [29,30].

As the name indicates, the EUROHIS-QOL-8 is a QoL index composed of eight items. From a conceptual point of view, each domain (physical, psychological, social relations, and environment) is represented by two items [31]. The result is a global index calculated from the sum of the eight items, whereby a higher score indicates a better perception of QoL. Cronbach α coefficient was 0.796 (0.584–0.907).

The B-IPQ is a generic nine-item questionnaire developed to assess the cognitive and emotional representations of disease. It consists of eight items assessing several illness perception dimensions: consequences, timeline, personal and treatment control, identity, understanding, and emotional response [32]. The higher the score on the B-IPQ, the more negative the patient’s illness perception. Cronbach α coefficient was 0.769 (0.462–0.821).

The Health Assessment Questionnaire disability index (HAQ-DI) assesses the degree of functional disability status in 8 categories: the ability to dress, get up, feed oneself, walk, perform personal hygiene, reach, grasp, and perform daily living activities [33]. Total scores close to 3 indicate more severe limitations and, therefore, less functional capacity. Cronbach α coefficient was 0.893 (0.813–0.943).

The BaSIQS questionnaire evaluates the quality of sleep through 7 items (events over the last week) on a scale of 0–5, where the total score may vary from 0–28 points [34]. Higher values indicate worse sleep quality. Cronbach α coefficient was 0.896 (0.826–0.942).

### 2.4. Determination of Circulating Concentration of Inflammatory Biomarkers

The assessment of the IL-6 circulating levels (serum) was performed by ELISA, using a human IL-6 ELISA Max™ Set Deluxe (BioLegend, Inc., San Diego, CA, USA), in accordance with the manufacturer’s instructions. Serum levels of CRP were determined using chemiluminescent microparticle immunoassay (CMIA) (Architect, Abbott Laboratory, Abbott Park, IL, USA), according to the manufacturer’s instructions. To avoid interassay variations, all samples were analyzed with the same kit on the same day.

### 2.5. Wearable Sensing Device

The sensing device, Xiaomi MI Band 4 (Xiaomi Inc., Beijing, China), was selected based on its previously reported utility, price, and average data recording time [35]. This wearable device allowed real-time data collection, including physiological indicators such as heart rate, step count, and sleep hours (classifying light sleep and deep sleep) as indicators of physical activity and quality of sleep. Patients were asked to continuously wear the smartband during the period of balneotherapy. Afterward, the recorded data were extracted into a tabular format (CSV) using the device integration API.

### 2.6. Statistical Analysis

Statistical analyses were performed using GraphPad Prism (GraphPad Software, La Jolla, CA, USA) and Stata, version 15 (StataCorp., College Station, TX, USA). Continuous variables data were expressed as the mean and SD. Student’s t-test or Wilcoxon test was used to compare changes in variables between the two groups, and a paired *t*-test was used to compare pre-and post-intervention variables within each group. The effect size using Cohen’s d has been estimated (small effect size—d = 0.2; medium effect size—d = 0.5; and large effect size—d = 0.8). The difference between means (pre- and post-balneotherapy) and 95% Confidence Interval (CI) was also estimated for statistically significant results. Significance was set at 5%, *p* ≤ 0.05.

## 3. Results

The baseline sociodemographic characteristics of the study population (*n* = 28) are shown in Table 2.

Table 3 shows the scores obtained in questionnaires. MD patients improved the QoL measured by 5Q-5D-5L (*p* < 0.001), EQ-VAS (*p* < 0.001), and EUROHIS-QOL (*p* = 0.017) after balneotherapy. Regarding illness perception, the participants had significantly lower B-IPQ sum scores (*p* < 0.001) after balneotherapy. The degree of functional capacity measured by HAQ-DI was also improved with balneotherapy (*p* = 0.019). The effect size varied from *d* = 0.581 to *d* = 0.980. The study group had a significantly lower BaSIQS (*p* = 0.019) score, revealing an improvement in the quality of sleep (Table 3).

**Figure 3:**
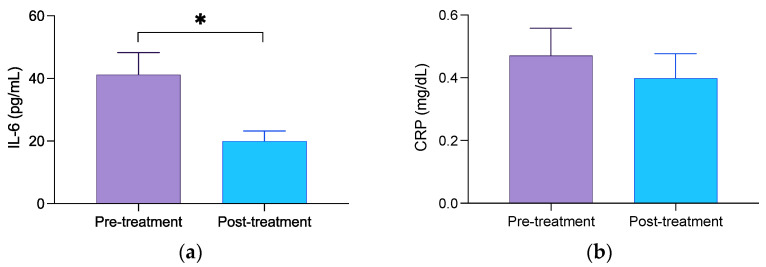
The serum concentration of IL-6 (**a**) and CRP (**b**) in MD patients before and after the balneotherapy. Columns represent the mean ± SD of independent assays performed in duplicate for each participant. * *p* < 0.05 concerning baseline values mean. IL-6 difference 95% CI; −21.286 (−31.026, −11.546).

Figure 3 shows the results relating to the effect of balneotherapy on circulating levels of regulatory cytokine levels, IL-6, and of systemic inflammation biomarker CRP. Serum concentrations of IL-6 (*p* < 0.001; Figure 3a) were markedly decreased after the 14-day balneotherapy. Regarding the circulating levels of CRP, a decreasing pattern was observed after the balneotherapy, although with no statistically significant difference (*p* = 0.174; Figure 3b). The effect size on circulating IL-6 was *d* = 0.847.

All participants used the smartband for 14 days. The data from activity, heart rate, and sleep are provided in Table 4. Despite not being statistically significant, the achieved results showed that most participants slept an average of 352.311 ± 56.664 min and 362.979 ± 53.320 min in different treatment time points, pre- and post-treatment for 14 days, respectively. Specifically, the hours of light sleep were greater than deep sleep.

## 4. Discussion

According to the literature, in order to evaluate and measure the potential benefits of therapeutic intervention, it is essential to conduct well-structured studies and adhere to a plan that follows the recommended practices in the field [1,2,3,4].

Balneotherapy is an approach whose perceived health-related impacts are not widely established in the scientific literature, being acknowledged as something that produces considerably positive effects from an intangible cultural and social heritage. This scarce evidence-based knowledge extends to the recognition of balneotherapy’s beneficial effects and the efficacy and accuracy of inherent therapeutic procedures [7,8,10].

Considering the current context and the growing acceptance of balneotherapy as a substitute for conventional medical treatment and a means of promoting health and wellness, it is crucial to conduct research that not only examines the effects of balneotherapy but also delves into the underlying therapeutic techniques and methodologies.

Therefore, in this study, we developed an open intervention trial focused on assessing the efficacy of the most common therapeutic approach used in thermal spas, which consists of a treatment plan of 14 consecutive days in which patients undergo daily treatments with natural mineral water specifically targeted to the category of pathology they suffer from. To establish the abovementioned efficacy, we have focused on patients suffering from MD pathologies and on balneotherapy’s ability to influence the inflammatory status, health-related QoL and quality of sleep, underlying overall health state, and clinically relevant benefits of the referred patients.

The concept of health-related QoL and its determinants have evolved to encompass those aspects of overall QoL that can be clearly shown to affect health—either physical or mental [36,37]. Sleep plays a vital role in an individual’s quality of life and well-being by regulating various cellular processes, cognitive function, energy storage, and disease prevention. Moreover, research suggests that both the duration and quality of sleep can have an impact on memory, learning, performance, and the metabolic and endocrine systems [38].

Overall, the patient’s perception of QoL, measured by three different instruments (5Q-eD-5L, EQ-VAS, and EUROHIS-QOL), was significantly improved after 14 days of balneotherapy. The potential effectiveness of this therapy was also corroborated by improvements in functional disability status (HAQ-DI) and illness perception (B-IPQ). Similarly, there was a statistically significant improvement in sleep quality BaSIQS score. These findings confirmed that balneotherapy is effective in managing MD, as reported by several previous studies [6,12,15,16,36,39], including a study from our group in the same spa center [40].

Balneotherapy appears to enhance the outlook for MD patients, as evidenced by the current study’s finding that circulating IL-6 levels fell after treatment and were higher than those earlier observed in healthy controls by Ortega et al. (2009) [41]. Similar findings were made by other studies, which discovered that mud treatment and balneotherapy significantly reduced systemic amounts of IL-6 as well as further important inflammation and regulatory cytokines [12,42]. IL-6 is known to affect both immune and non-immune system cells, and it often exhibits hormone-like properties that influence homeostatic functions. Clinical studies have established a correlation between elevated levels of IL-6 and heightened pain in individuals suffering from MD, such as rheumatoid arthritis and fibromyalgia [10].

One of the first cytokines to be triggered in the signaling system during an inflammatory process is IL-6, as well. Subsequently, a systemic acute-phase reaction occurs, which leads to the production of significant amounts of acute-phase proteins originating from hepatocytes, such as CRP. After 14 days of balneotherapy, no statistically significant change in circulating CRP levels was found in our research. The amounts of this protein did, however, appear to be dropping. Numerous studies have shown, as summarized by Gálvez et al. (2018) [10], that individuals with rheumatic, cardiovascular, and diabetes diseases have lower CRP levels following balneotherapy [42,43,44,45].

The mechanism underlying the health-improving effects of thermal therapies, including balneotherapy, are not fully clear. However, in recent decades, numerous studies have shed some light on the immunological mechanisms of effectiveness, highlighting anti-inflammatory effects, pain relief, and improvement of overall health status as some of the bases of balneotherapy’s clinical benefits [10,19,20,21,22]. The benefits of balneotherapy are believed to stem from a complex and synergistic combination of various factors, including mechanical, thermal, and chemical elements. These physiological responses primarily involve neuroendocrine and immunological effects induced by sensory surfaces, such as the skin and mucous membranes [46,47]. Several theories have been proposed, including endogenous opioids, adrenal axis activation, reduced circulating levels of some inflammatory mediators, increased circulating levels of cortisol and endorphin, and the elevation in sympathovagal balance [17,47]. Furthermore, the equilibrium between the increased elasticity of tissues rich in collagen and the decreased spasm in muscles and the increase in the pain threshold and improvement of the joints’ function has also been proposed [17,39,43,47,48]. Recently, Carbajo and Maraver (2018) suggested that salt mineral waters act via a cell osmosis mechanism conditioned by salt characteristics and concentration, which can activate/inhibit cell apoptosis and necrosis. As a result, the mechanosensitive piezoelectric channels are modulated by the osmotic mechanism, which affects bodily functions such as somatosensation, red blood cell volume regulation, and blood vessel physiology [49].

We used a wearable device for real-time patient monitoring during the balneotherapy treatment period. Although no statistical associations were found in the heart rate, step count, and sleep hours results, the smartband was able to record the pretended data, even with the extreme conditions towards electronic devices that typically exist in natural mineral water-based spas. After extracting the data collected by the smartbands during the 14 days of treatment, we were able to acknowledge that the used smartband (*Xiaomi MI Band 4*) was still able to collect valid and reliable data, thus ensuring alignment with the arguments presented by Pérez et al. [35] study in which these authors demonstrate the referred smartband had reasonable accuracy and precision and allows monitoring of the average step count and heart rate in free-living conditions, indoor and outdoor. Furthermore, researchers are increasingly utilizing smartbands with sleep-tracking capabilities (like the one employed in this study) due to their ability to non-invasively monitor sleep patterns [50]. Additionally, other authors found that this type of device could be an objective tool for assessing sleep patterns in adults and older adults and determining its impact on health status and QoL [51,52].

The major limitation of this study was the clinical trial design as open-label. The sample size and the lack of a control group may also be listed among the most significant limitations. Further studies on larger patient groups with longer follow-up periods are necessary to strengthen these results. A randomized controlled trial would increase confidence in interpreting the effects of the intervention.

## 5. Conclusions

The present study shows that balneotherapy might be considered an alternative treatment for managing the health status of MD patients. The results suggest that the clinical benefits of balneotherapy may be mediated by an anti-inflammatory effect in MD patients. A decrease in the circulating levels of IL-6 was observed, along with positive contributions to reducing pain, the functionality of patients, QoL, quality of sleep, and disability perception status. The Cronbach α coefficient confirmed the good internal consistency of the instruments.

To the best of our knowledge and from a conceptual standpoint, there is no other method available that employs a multidisciplinary approach to evaluate the efficacy of balneotherapy in treating MD pathologies. As a result, this research has the potential to provide valuable insights for those involved in the study of natural mineral water.

## Figures and Tables

**Table 2:** Baseline sociodemographic characteristics of the study population.

	Total (*n* = 28)
**Age** (mean ± SD)	56.07 ± 9.641
**Gender, *n* (%)**	
Male	6 (21.4)
Female	22 (78.6)
**Marital status, *n* (%)**	
Single	2 (7.2)
Married/Consensual union	22 (78.6)
Divorced/widower	4 (14.2)
**Education, *n* (%)**	
Less than high school	12 (42.9)
High school	6 (21.4)
Graduated	10 (35.7)
**Employment status, *n* (%)**	
Unemployed	1 (3.7)
Pensioner/Retired	5 (17.9)
Employed	17 (60.7)
Self-employed	5 (17.9)
**Private household, *n* (%)**	
1	2 (7.1)
2	14 (50.0)
3	10 (35.8)
4	2 (7.1)

**Table 3:** Scales’ scores recorded in pre- and post-balneotherapy.

Instruments	Pre-Treatment	Post-Treatment	Difference (95% CI)	Effect Size (*d*)	*p*-Value
5Q-5D-5L	0.780 (±0.236)	0.917 (±0.114)	0.137 (0.062, 0.208)	0.980	<0.001
EQ-VAS	67.857 (±17.020)	81.786 (±10.560)	13.929 (7.081, 20.775)	0.789	<0.001
EUROHIS-QOL	68.192 (±12.149)	72.545 (±9.711)	4.353 (0.842, 7.864)	0.581	0.017
B-IPQ	37.536 (±6.010)	28.643 (±4.653)	−8.893 (−13.209, −4.577)	0.799	<0.001
BaSIQS	13.071 (±8.750)	9.107 (±9.708)	−3.964 (−5.769, −2.159)	0.852	<0.001
HAQ-DI	0.268 (±0.431)	0.112 (±0.305)	−0.156 (−0.292, −0.021)	0.747	0.019

Data are expressed as mean (±SD); 95% Confidence Interval (CI).

**Table 4:** Smartband data was recorded in pre (week-1)- and post (week-2)-balneotherapy.

	Week-1	Week-2	*p*-Value
Number of steps	9900.541 (±4276.198)	9498.555 (±4508.623)	0.399
Heart rate	72.172 (±6.316)	71.146 (±6.243)	0.557
Deep sleep (minutes)	93.714 (±34.961)	99.429 (±38.159)	0.290
Light sleep (minutes)	352.311 (±56.664)	362.979 (±53.320)	0.225

Data are expressed as mean (±SD).

## Data Availability

The data presented in this study are available on request from the corresponding author. The data are not publicly available due to GDPR compliance.
